# Neutrophil-to-lymphocyte ratio is a predictive marker for anti-MDA5 positive dermatomyositis

**DOI:** 10.1186/s12890-022-02106-8

**Published:** 2022-08-17

**Authors:** Tao Liu, Wen Li, Zehao Zhang, Ting Jiang, Yu Fei, Jing Huang, Qibing Xie

**Affiliations:** 1 Department of Rheumatology and Immunology, West China Hospital, Sichuan University, Chengdu, 610041 Sichuan People’s Republic of China; 2 West China School of Public Health/West China Fourth Hospital, Sichuan University, Chengdu, 610041 Sichuan People’s Republic of China

**Keywords:** Anti-MDA5, Dermatomyositis, Neutrophil-to-lymphocyte ratio

## Abstract

**Background:**

NLR is a systemic inflammatory marker that have been associated with overall survival in patients with some rapidly progressive disease. There are few data about the diagnostic and predictive value of NLR in autoimmune diseases, and it has not been described in anti-MDA5 positive DM. We try to correlate neutrophil-to-lymphocyte ratio (NLR) with fatality from dermatomyositis in anti-MDA5 positive patients.

**Method:**

A retrospective study in which 195 patients were enrolled was conducted. Clinical and laboratory information was collated and ratios of neutrophil to lymphocyte counts (NLR) calculated. The primary end point was all-cause death.

**Result:**

Of the 195 patients studied, all had interstitial lung disease, including 140 survivors and 55 non-survivors. An optimal NLR cut-off value of 4.86 for mortality prediction was identified. The NLR of non-survivors was significantly higher than that of survivors (*p* < 0.001). Plasma levels of lactate dehydrogenase (LDH) and C-reactive protein were significantly increased when NLR was greater than 4.86. Results of multivariate analysis established that NLR > 4.86 was an independent predictor of mortality (HR: 2.52; 95%CI: 1.33–4.78; *p* = 0.005). Abstinence from smoking (HR: 2.66; 95%CI: 1.33–4.78; *p* = 0.003), emergence of rapidly progressive interstitial lung disease (RPILD; HR: 4.38; 95%CI: 2.37–8.08; *p* < 0.001), low plasma LDH (HR: 3.82; 95%CI: 2.06–7.11; *p* < 0.001) and presentation with dyspnea (HR: 2.17; 95%CI: 1.22–3.86; *p* = 0.009) were all protective factors predictive of survival.

**Conclusion:**

NLR is a cost-effective and widely accessible biomarker with utility for risk stratification in patients with anti-MDA5 + dermatomyositis.

**Supplementary Information:**

The online version contains supplementary material available at 10.1186/s12890-022-02106-8.

## Introduction

Anti-MDA5 + dermatomyositis (DM) is an idiopathic inflammatory myopathy (IIM) which commonly presents with skin manifestations and progresses to pulmonary involvement. The involvement of muscle tissue is relatively rare. Patients testing positive for anti-MDA5 account for 13–30% of all IIM [1]. During inflammatory activation, patients are prone to rapidly progressive pulmonary interstitial lesions which result in high mortality. Vascular inflammation is a dominant part of inflammatory activation and peripheral blood neutrophils and lymphocytes participate in the process [2]. The ratio of neutrophil to lymphocyte counts (NLR) is an indicator of systemic inflammation which is easier to obtain than other inflammatory indicators, such as plasma lactate dehydrogenase (LDH) or ferritin. Levels of LDH [3] and ferritin [4, 5] are well-established as good prognostic indicators, allowing correlations to be made between the severity of the disease and survival time. To date, little data regarding the diagnostic and predictive value of NLR in autoimmune diseases has been reported and any utility for anti-MDA5 + DM has not been evaluated. Therefore, the current study aimed to determine whether NLR has value for mortality prediction in cases of anti-MDA5 + DM.

## Methods

### Patient enrollment

Patients receiving treatment for anti-MDA5 + DM in West China Hospital, Sichuan, China between December 2015 and September 2021 were retrospectively recruited. The study was approved by the bioethics committee of West China Hospital (NO.246 in 2019). Anti-MDA5 + DM was diagnosed according to the guidelines published by the 239^th^ European Neuro Muscular Center [6]. Interstitial lung disease (ILD) diagnosis relies on symptoms, signs and high-resolution CT (HRCT) [7]. Rapidly progressing ILD (RP-ILD) was defined according to the criteria proposed by Akira et al. [8] and HRCT score evaluated by 2 independent radiologists, according to the method of Ichikado et al. [9]. All patients were given unique ID numbers on enrollment. Some patients were regularly followed up in our hospital and patient survival status was confirmed from the electronic medical record. Other patients were not regularly followed up and families were contacted for survival information, including time and cause of death. Neutrophil and lymphocyte counts were recorded on the patient’s first admission. NLR was defined as absolute neutrophil count divided by absolute lymphocyte count. Plasma levels of creatine kinase (CK), LDH and C-reactive peptide (CRP) were recorded, along with other clinical symptoms. Titers of anti-MDA5 antibodies were analyzed by transfection (Shaanxi MY Biotech Co., Ltd.).

## Statistical analysis

All data analysis was performed using R statistical programming package, version 4.1.3 (R Programming). Overall survival (OS) was calculated from first treatment to death (event) or to last follow-up (census). The Kolmogorov–Smirnov test was used to assess the normality and homogeneity of variance of all the data. Data are presented as mean ± standard deviation when normally distributed or median and interquartile range (IQR) for non-normally distributed continuous variables and numbers (percentages) for categorical variables. Univariate and multivariate COX regression analyses were performed to identify independent risk factors with an impact on OS. OS curves and comparisons were calculated by Kaplan–Meier survival curves and the log-rank test. The optimal truncation value of NLR affecting prognosis was determined by receiver operator characteristic (ROC) curve in R Programming. All statistical tests were two sided and a *p* < 0.05 considered statistically significant.

## Results

**Fig. 1:**
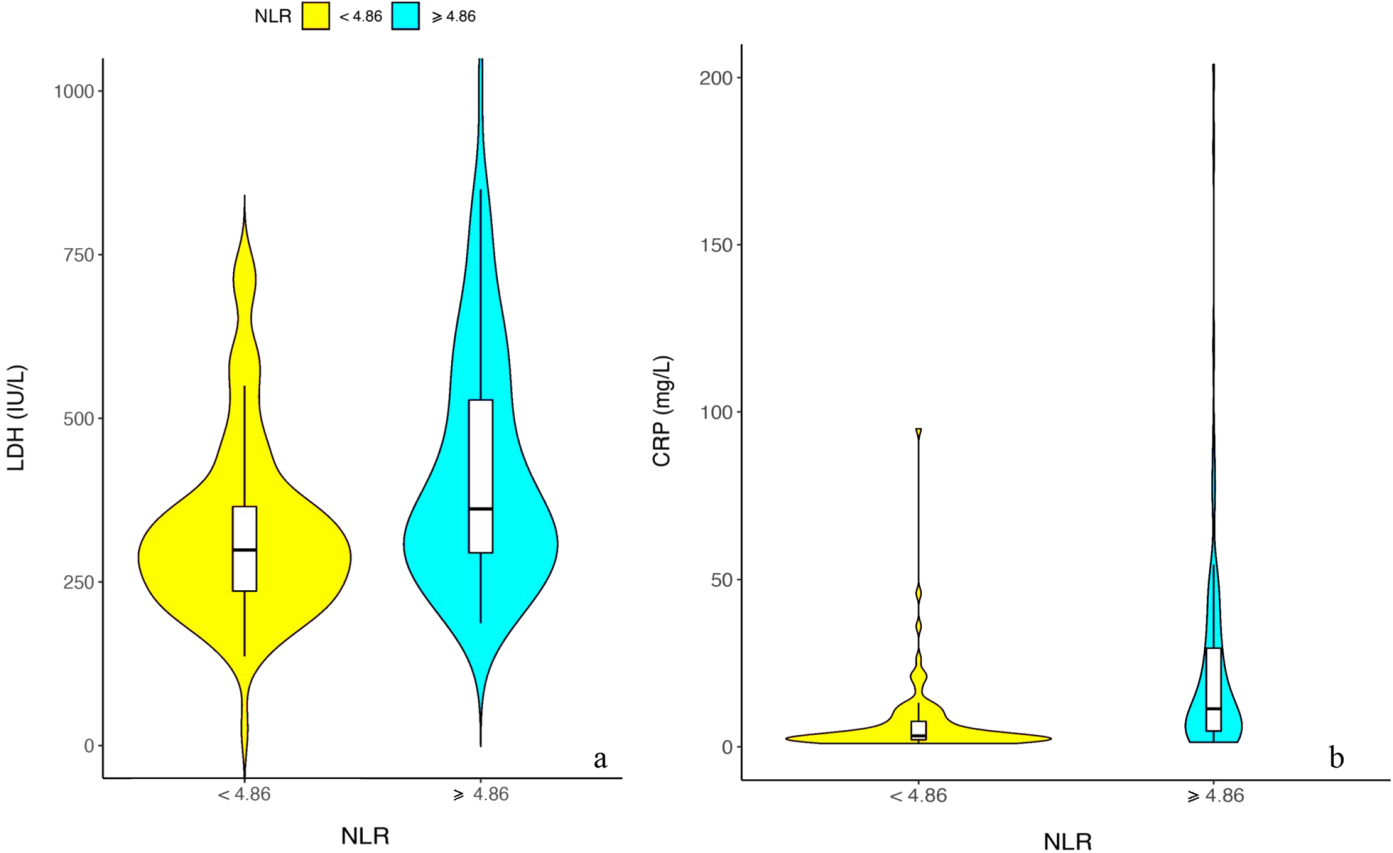
Median and interquartile range of LDH (**a**) and CRP (**b**) related to NLR. *Abbreviation*: LDH: lactate dehydrogenase; CRP: C-reactive protein; NLR: neutrophils/lymphocyte ratio

A total of 234 anti-MDA5 + DM patients were identified during the period between December 2015 and September 2021. Of the total, 4 patients were excluded due to missing core data and 35 patients presented without ILD on first admission and were excluded on the grounds that patients without ILD had a better prognosis than those with ILD. After these exclusions, 195 patients remained and were enrolled. There were 31 patients who did not receive corticosteroids at baseline for whom NLR data were obtained: 164 patients received corticosteroids. Baseline patient characteristics are shown in Table 1. The median value for months of the observational period was 26.5 (95% CI: 21.2–29.3). The mean age of the cohort was 50.34 ± 10.56 years. Two thirds (68.21%) were female, and the majority (83.59%) had never smoked. The mean survival time of the NLR ≥ 4.86 group was 58.85 months and that of the NLR ≤ 4.86 group was 34.41 months, *p* < 0.001. Some patients had comorbidities, such as heart failure (8.72%), diabetes (8.72), fatty liver (20.00%) and hypertension (7.18%), although these comorbidities appeared to have little impact on prognosis. More than half of the patients had arthralgia (61.54%), Gottron sign (61.54%) or heliotrope sign (51,79%). Almost half had developed dyspnea by the first admission (42.05%). Skin ulcers were relatively rare (11.79%). The median survival time was 13.50 months (1.64–32.56 months). Levels of inflammatory biomarkers, such as LDH (*p* < 0.001), CRP (*p* < 0.001) and red blood cell distribution width (RDW) (*p* = 0.025), showed significant differences between survivors and non-survivors. Non-survivors had higher CK levels than survivors (80.00 IU/L vs 51.00 IU/L; *p* = 0.002). Median values for white blood cell and platelet counts were 5.59 × 10^9^/L (4.06–7.44 × 10^9^/L) and 195.22 ± 71.36 × 10^9^/L, respectively. The median NLR value was 4.85 (3.41–7.07) with lower values in anti-MDA5 + survivors than in non-survivors (4.34 vs 6.19; *p* < 0.001). Changes in NLR were synchronized with those of other inflammatory markers, such as LDH (Fig. 1a) and CRP (Fig. 1b).

**Table 1:** Demographic and clinical characteristics of different groups of anti-MDA5 + patients with ILD

	Overall	Survival	Un-survival	*p*
*n*	195	140	55	
Age (mean ± SD)	50.34 ± 10.56	49.38 ± 10.56	52.78 ± 10.24	0.038
Gender (male %)	62 (31.79)	41 (29.29)	21 (38.18)	0.303
Smoking (%)	32 (16.41)	18 (12.86)	14 (25.45)	0.055
Survival time (median [IQR])	13.50 [1.64, 32.56]	24.13 [8.29, 40.02]	0.77 [0.33, 2.50]	< 0.001
*Clinical signs and symptoms*				
Heart failure (%)	17 (8.72)	9 (6.43)	8 (14.55)	0.127
Diabetes (%)	17 (8.72)	13 (9.29)	4 (7.27)	0.868
Fatty liver (%)	39 (20.00)	30 (21.43)	9 (16.36)	0.551
Hypertension (%)	14 (7.18)	7 (5.00)	7 (12.73)	0.116
Arthralgia (%)	120 (61.54)	91 (65.00)	29 (52.73)	0.155
Dyspnea (%)	82 (42.05)	49 (35.00)	33 (60.00)	0.003
Skin ulcer (%)	23 (11.79)	19 (13.57)	4 (7.27)	0.327
Gottron sign (%)	120 (61.54)	87 (62.14)	33 (60.00)	0.910
Heliotrope sign (%)	101 (51.79)	79 (56.43)	22 (40.00)	0.057
HRCT score (median [IQR])	84.00 57.00, 117.00]	77.00 [54.75, 108.75.75]	99.00 [70.00, 137.00]	0.001
RPILD (%)	63 (32.31)	28 (20.00)	35 (63.64)	< 0.001
*Laboratory examination*				
RDW (median [IQR])L/L	47.60 [44.80, 52.30]	47.00 [44.20, 51.80]	49.20 [46.15, 54.20]	0.025
CK (median [IQR]) IU/L	56.00 [31.50, 120.00]	51.00 [30.00, 99.00]	80.00 [45.50, 220.50]	0.002
CRP (median [IQR]) (mg/L)	5.87 [2.54, 16.75]	4.27 [2.40, 10.48]	15.30 [4.75, 34.40]	< 0.001
LDH (median [IQR]) IU/L	321.00 [261.50, 441.50]	301.00 [232.50, 365.25]	443.00 [327.00, 658.00]	< 0.001
WBC (median [IQR])10^9^/L	5.59 [4.06, 7.44]	5.42 [3.86, 7.06]	6.47 [4.33, 8.63]	0.066
PLT (mean ± SD) 10^9^/L	195.22 ± 71.39	197.66 ± 65.18	189.00 ± 85.55	0.174
Neutrophils (median [IQR]) 10^9^/L	4.27 [2.85, 5.74]	4.03 [2.78, 7.18]	4.81 [3.20 6.97]	0.032
Lymphocytes (median [IQR]) 10^9^/L	0.79 [0.57, 1.09]	0.88 [0.65, 1.12]	0.72 [0.46, 0.97]	0.008
NLR (median [IQR])	4.85 [3.41, 7.07]	4.34 [3.15, 6.63]	6.19 [4.70, 9.84]	< 0.001

Data are presented as numbers (percentages), mean ± standard deviation (± SD) or median values and interquartile range (IQR)

*RDW* red cell volume distribution width; *CK* creatine kinase; *CRP* C-reactive protein; *LDH* lactate dehydrogenase; *WBC* white blood cell count; *PLT* platelet count; *NLR* neutrophils/lymphocyte ratio; *DM* dermatomyositis; *RPILD* rapidly progressive interstitial lung disease; *HRCT* high-resolution CT; *LDH*: lactate dehydrogenase

### Univariate and multivariate analysis

Results of univariate analysis showed that NLR (*p* < 0.001), plasma LDH (*p* < 0.001), smoking (*p* < 0.009), RPILD (*p* < 0.001), dyspnea (*p* < 0.001) and HRCT score were associated with OS of MDA5 + DM patients (Table 2).

**Table 2:** Variables associated with death among patients with anti-MDA5 + DM from Univariable Cox Regression Analysis

Characteristics	HR (95%CI)	*P* Value
Gender	1.48 (0.86–2.55)	0.16
Age	1.13 (0.57–2.25)	0.717
Smoking	2.25 (1.22–4.12)	0.009
Arthralgia	0.65 (0.38–1.1)	0.106
Dyspnea	2.62 (1.52–4.5)	< 0.001
Fatty liver	0.78 (0.38–1.58)	0.486
Dysphagia	0.52 (0.13–2.12)	0.36
Diabetes	0.76 (0.28–2.11)	0.602
Hypertension	1.84 (0.83–4.07)	0.132
Infection	3.01 (1.52–5.98)	0.002
Skin ulcer	0.51 (0.18–1.41)	0.192
Gottron sign	0.88 (0.52–1.52)	0.656
Heliotrope sign	0.57 (0.33–0.97)	0.039
RPILD	6.46 (3.69–11.3)	< 0.001
HRCT score	3.62 (1.99–6.6)	< 0.001
LDH (IU/L)	5.28 (2.94–9.48)	< 0.001
Lymphocytes (10^9^/L)	0.51 (0.27–0.94)	0.032
CRP (mg/L)	1.01 (1.01–1.02)	< 0.001
NLR	3.93 (2.14–7.22)	< 0.001

*HR* hazards ratio; *CI* confidence interval

**Fig. 2:**
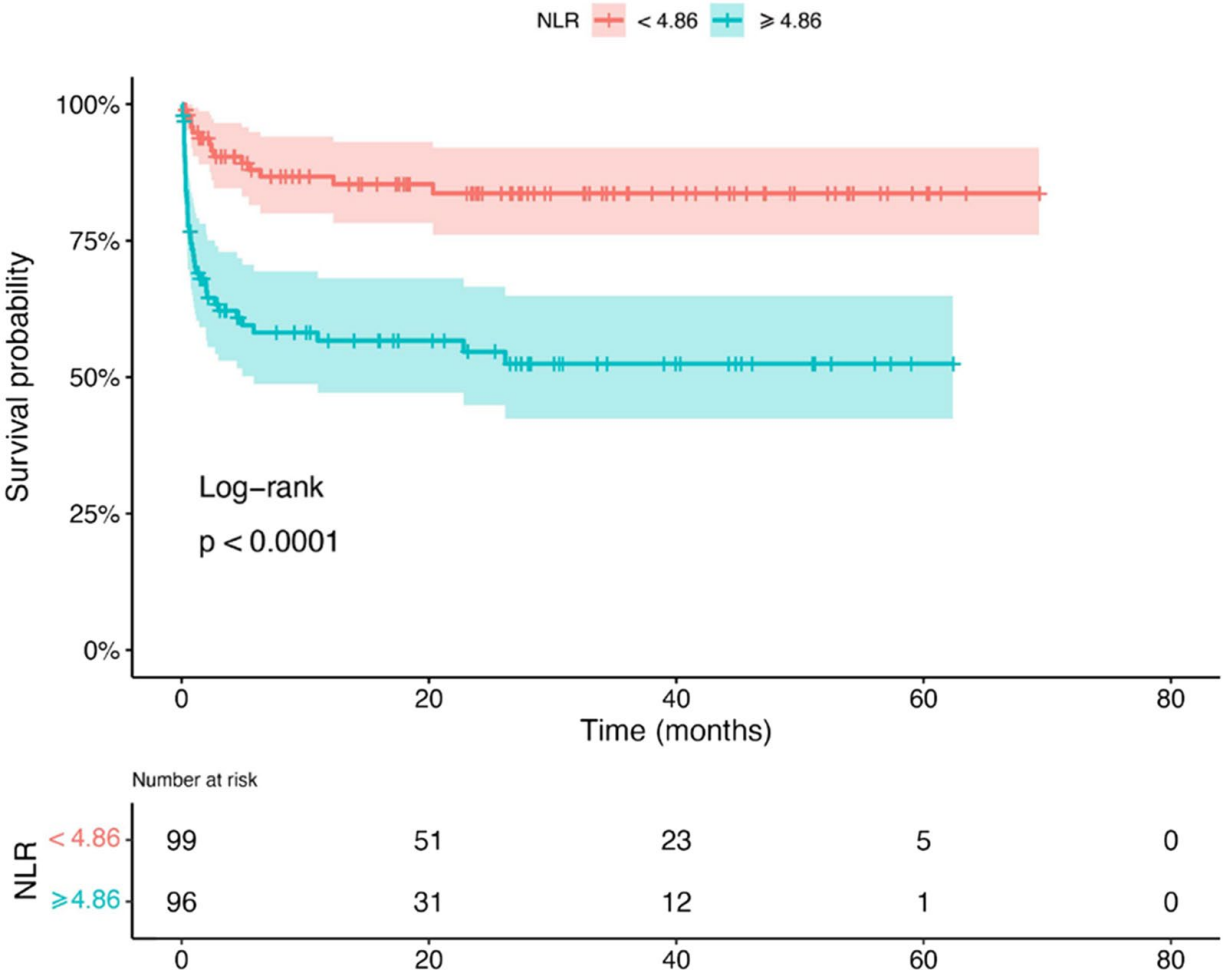
Survival curve of anti-MDA5 + DM patients with ILD based on initial NLR. The survival rate was calculated by the Kaplan-meier test and compared using the log-rank test. *Abbreviation*: NLR: neutrophils/lymphocyte ratio

Factors identified by univariate analysis as being related to survival were subjected to multivariate COX regression analysis. Neutrophil count, platelet count, creatine kinase and white blood cell count were omitted from data presented from Cox univariate regression analysis because *p* ≥ 0.05. The optical truncation value of NLR affecting the prognosis was found to be 4.86 by R statistical analysis (Additional file 1: Fig. S1). The Youden index was 0.37, the sensitivity was 0.74, the specificity was 0.62 and the positive and negative predictive values were 0.43 and 0.859. Kaplan–Meier curves showed that patients with a lower NLR (< 4.86) at baseline showed significantly higher OS compared with those with a higher NLR (≥ 4.86) at baseline (*p* < 0.001; Fig. 2).

**Fig. 3:**
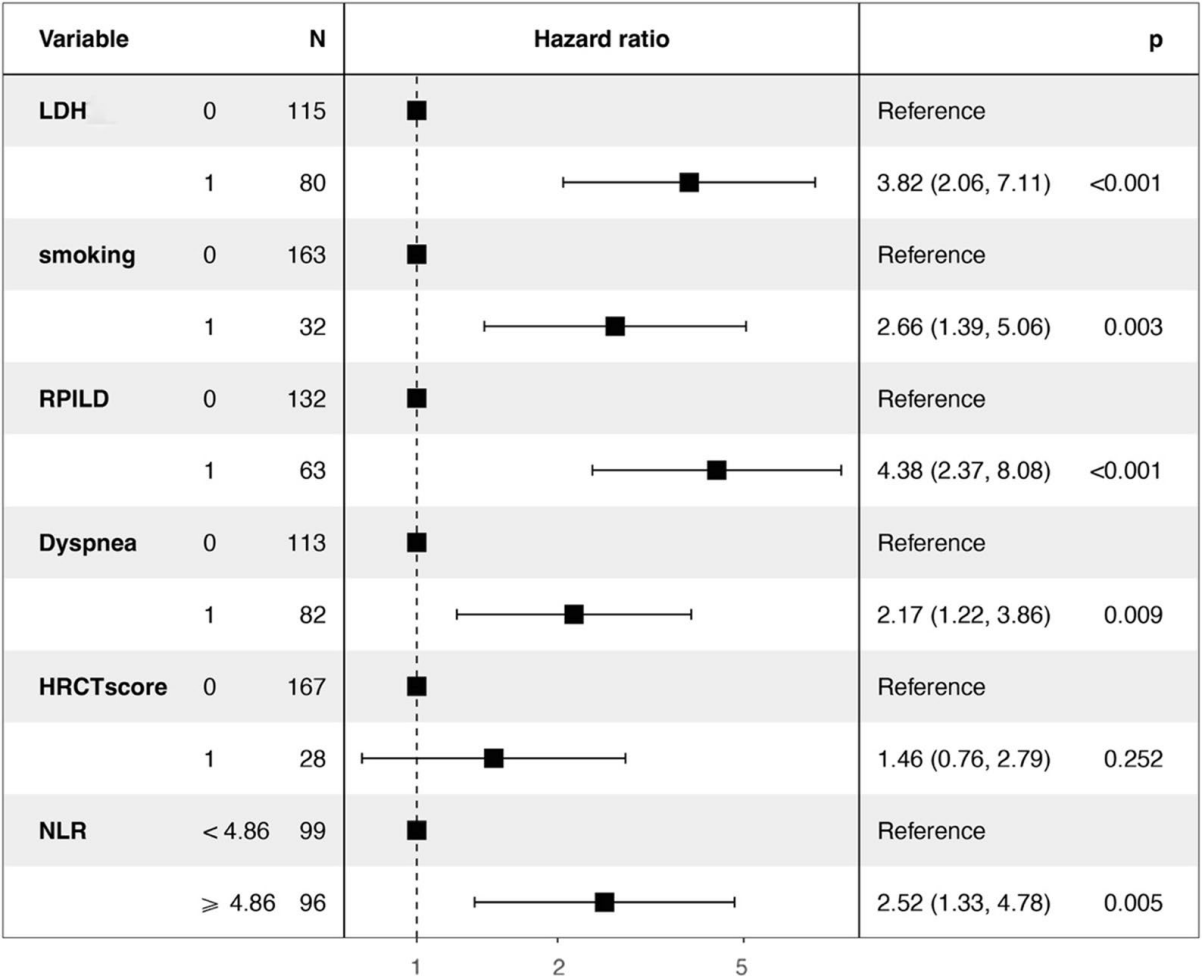
Forest plot of the multivariable cox analysis of the prognostic factors for patients with ILD in anti-MDA5 + DM of the discovery cohort. The  indicates the weight of the variable in the multivariable cox analysis; higher number indicates greater weight; variables with *P *< 0.05 in the univariate analysis were included in the multivariable analysis.* Abbreviation*: LDH: lactate dehydrogenase; RPILD: rapidly progressive interstitial lung disease; HRCT: high-resolution CT; NLR: neutrophils/lymphocyte ratio

Patients with a history of smoking had a higher risk of death than non-smokers (HR: 2.66; 95%CI: 1.33–4.78; *p* = 0.003). The prognosis of patients with RPILD was poorer than patients without RPILD (HR: 4.38; 95%CI: 2.37–8.08; *p* < 0.001). Levels of plasma LDH (HR: 3.82; 95%CI: 2.06–7.11; *p* < 0.001) and dyspnea (HR: 2.17; 95%CI: 1.22–3.86; *p* = 0.009) were predictive of survival. However, HRCT score was not a prognostic indicator for anti-MDA5 + DM patients (Table 3). A forest plot for subgroup analyses of overall survival is presented in Fig. 3.

**Table 3:** Variables associated with death among patients with anti-MDA5 + DM from Multivariable Cox Regression Analysis

Characteristics	HR (95% CI)	*P* Value
LDH(IU/L)	3.82 (2.06–7.11)	< 0.001
Smoking	2.66 (1.39–5.06)	0.003
RPILD	4.38 (2.37–8.08)	< 0.001
Dyspnea	2.17 (1.22–3.86)	0.009
HRCT score	1.46 (0.76–2.79)	0.252
NLR ≥ 4.86	2.52 (1.33–4.78)	0.005

*HR* hazards ratio; *CI* confidence interval

## Discussion

DM is a heterogeneous autoimmune disease with the anti-MDA5 + form exhibiting a characteristic rash and interstitial lung disease (ILD). The involvement of muscle tissue is rare, so that serum CK levels are usually normal. However, many patients develop rapidly progressive acute pulmonary failure, accounting for high mortality rates [10].

Neutrophils and lymphocytes play important roles in systemic autoimmune diseases with numbers and functions changing during disease progression. A component of the routine blood cell examination (RBC), NLR values have attracted increased attention in recent years for their utility in inflammatory and autoimmune disease. Previous work has indicated that NLR reflects disease activity in rheumatoid arthritis (RA) [11] and Bechet disease (BD) [12, 13], is a predictive marker for psoriatic arthritis (PsA) [14] and relates to the occurrence of lupus nephritis in systemic lupus erythematosus (SLE) [15, 16]. However, to the best of our knowledge, the relationship between NLR and MDA5 + DM has not been previously studied. Multiple indicators have been correlated with the prognosis of anti-MDA5 + DM, such as anti-MDA5 tilter [17], ferritin [4, 5, 17, 18], KL-6 [19] and the proportion of CD4 + CXCR4 + T cells [20]. The current study revealed elevated NLR to be an independent predictor for poor survival in anti-MDA5 + DM patients, in addition to LDH, CRP and other inflammatory indicators.

Knowledge regarding the pathogenic mechanisms of DM remains limited but it seems to disproportionately affect genetically susceptible populations and is triggered by infectious agents (viruses, picornaviruses, flaviviruses) [2]. Neutrophils and lymphocytes produce a variety of cytokines and participate in DM pathogenesis. Vasculopathy is a well-established feature of MDA 5 + DM [21, 22]. Oxidative stress is involved in the pathophysiology of vascular inflammation in DM [23, 24]. Oxidative stress is associated with excessive inflammatory activity and NLR is a non-specific indicator of oxidative stress, reflecting the state of the body’s immune system [25, 26]. Antigen-stimulated responses in autoimmune diseases include production of reactive oxygen species and the resulting oxidative stress has an impact on disease progression, response to therapy and prognosis. NLR correlates with inflammatory factors, such as CRP, LDH and ferritin, and interplay of multiple factors, including pro- and anti-inflammatory factors, may be responsible for measured NLRs. NLR is also related to other pathological conditions, such as cancer [27–29], osteoarthritis [30, 31] and myocardial infarctions [32, 33].

NLR measurements are relatively inexpensive and easily incorporated into routine clinical practice. The predictive properties of NLR allow it to serve as a prognostic marker to aid clinical decision-making at an early stage of anti-MDA5 + DM disease. Anti-MDA5 + DM has a high mortality rate due to the common development of rapidly progressive interstitial lung disease (RP-ILD) which is difficult to treat, especially in combination with infection [34–36]. Seasonal and geographical variations in anti-MDA5 + DM suggest that infections, especially viruses, may be a predisposing factor, perhaps due to the induction of a cytokine storm [37, 38]. Viral RNA activates MDA5 in infected cells, leading to the production of type I interferon (IFN-I) and cytokines [38]. Increased neutrophils during bacterial infection and decreased lymphocytes during viral infection contribute to high NLRs and dismal prognoses. Intervention at the early stage of anti-MDA5 + DM, when elevated NLRs may first be detected, may prevent or delay the development of cytokine storms and tissue damage.

Plasma LDH levels have previously been reported to be increased in RPILD and associated with high titers of anti-MDA5 antibody. A recent study has suggested that LDH > 335/L was an independent risk factor for poor prognosis in anti-MDA5 + DM [3]. The current study found that serum LDH in patients with NLR > 4.86 was significantly higher than that in patients with NLR < 4.86 (Fig. 1a). This suggests that NLR measurements have a related function to those of LDH. However, LDH is released by many tissues, such as liver and kidney, and is greatly affected by CK levels. Similarly, CRP is a general reflection of inflammation that has been widely validated clinically with higher initial CRP levels significantly associated with both RPILD and poor outcome in anti-MDA5 positive patients[39]. Consistent with these findings, our study demonstrated that serum CRP was significantly higher in patients with NLR > 4.86 (Fig. 1b). Therefore, NLR may prove to be a more appropriate indicator of inflammatory state in anti-MDA5 + DM.

Previous studies have identified HRCT score as an independent risk factor for poor prognosis in anti-MDA5 + DM [5] but the current studies do not replicate those results. This inconsistency may have arisen due to selection of first admission HRCT images from patients who have been hospitalized and scanned on several occasions.

Some DM patients deteriorate rapidly, often within the time-scale of 1 month. Therefore, early HRCT scores may not reliably indicate abnormality and vigilance is required to ensure repeated scans on follow-up.

We acknowledge several limitations to the current study. All data were derived from patients presenting at a single center. We did not include ferritin in further analysis because nearly 50% of the ferritin values in our data set were missing, a deficiency that cannot be compensated by statistical methods. Moreover, NLR measured at initial presentation was included but not that after treatment, so that NLR changes could not be assessed. In addition, many factors, including treatment program and inflammatory severity, contribute to poor prognosis in anti-MDA5 + DM. Prognosis must be evaluated in combination with other indicators.

## Conclusion

In conclusion, NLR less than 4.86 was found to be an independent predictor of longer survival for patients with MDA5 + DM. NLR may prove to be a marker with clinical utility due to its low cost, accessibility and reproducibility.

## Supplementary Information

**Additional file 1. Figure S1**: Receiver operator characteristic curves for predicting non-survival between NLR ≤ 4.86 and NLR > 4.86 in anti-melanoma differentiation-associated gene 5 (anti-MDA5) antibody-positive dermatomyositis.

## Data Availability

The datasets generated and/or analyzed during the current study are not publicly available due to ethical/legal/commercial reasons but are available from the corresponding author on reasonable request.
