# Supplementary material for: Neutrophil-to-lymphocyte ratio is a predictive marker for anti-MDA5 positive dermatomyositis
Source: BMC Pulm Med. 2022 Aug 17;22:316. doi: 10.1186/s12890-022-02106-8 (PMC9382756; doi:10.1186/s12890-022-02106-8)
Supplement: Supplementary file 1 — Additional file 1. Figure S1: Receiver operator characteristic curves for predicting non-survival between NLR ≤ 4.86 and NLR > 4.86 in anti-melanoma differentiation-associated gene 5 (anti-MDA5) antibody-positive dermatomyositis. [file 12890_2022_2106_MOESM1_ESM.pptx]

## Slide 1
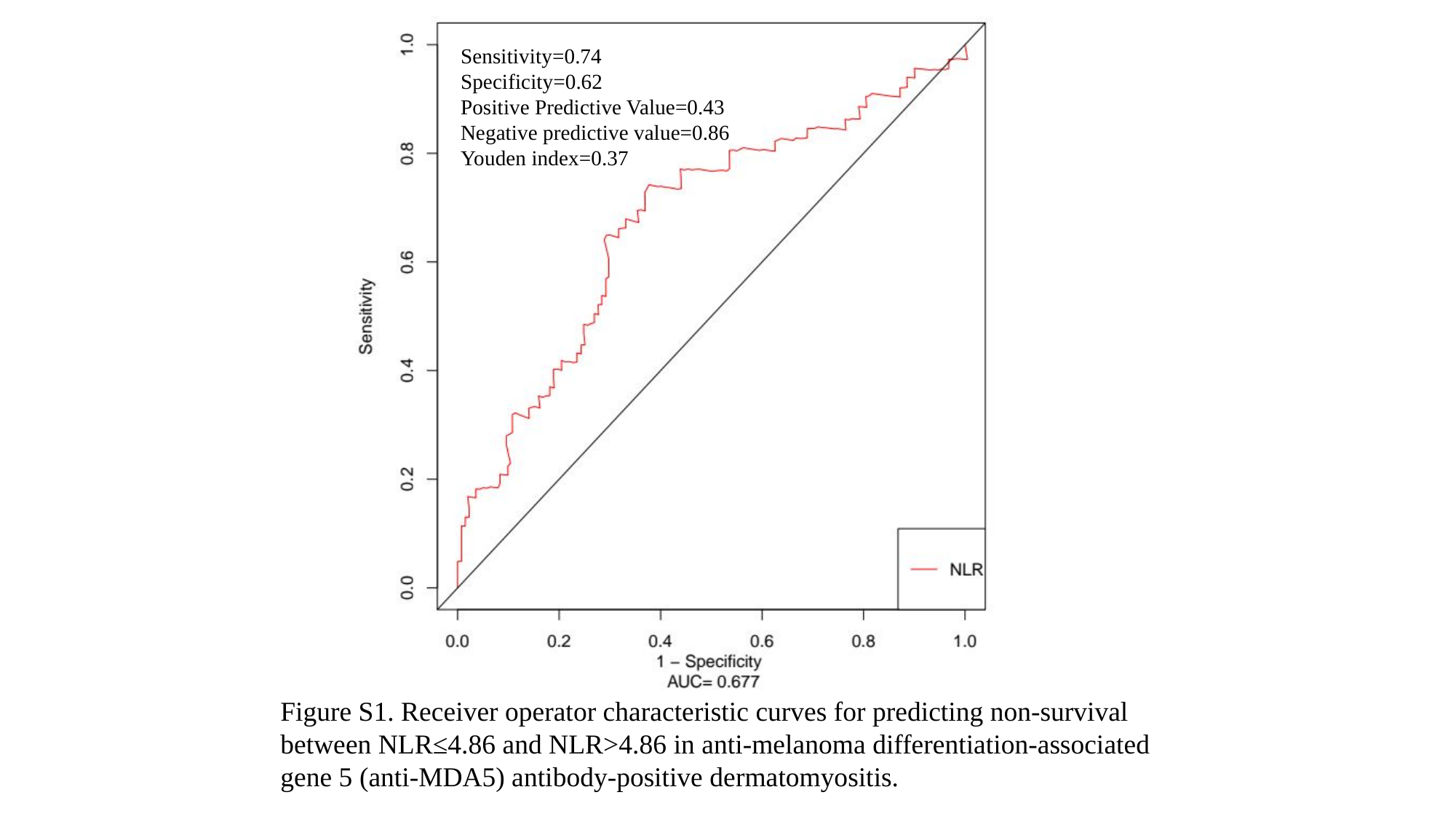

Sensitivity=0.74
Specificity=0.62
Positive Predictive Value=0.43
Negative predictive value=0.86
Youden index=0.37
Figure S1. Receiver operator characteristic curves for predicting non-survival between NLR≤4.86 and NLR>4.86 in anti-melanoma differentiation-associated gene 5 (anti-MDA5) antibody-positive dermatomyositis.
